# The Relationships between Perceived Design Intensity, Preference, Restorativeness and Eye Movements in Designed Urban Green Space

**DOI:** 10.3390/ijerph182010944

**Published:** 2021-10-18

**Authors:** Yu Wu, Zhixiong Zhuo, Qunyue Liu, Kunyong Yu, Qitang Huang, Jian Liu

**Affiliations:** 1 College of Landscape Architecture, Fujian Agriculture and Forestry University, Fuzhou 350000, China; 2201775003@fafu.edu.cn; 2 Xiamen University Tan Kah Kee College, Zhangzhou 363105, China; fjzzx@xujc.com; 3 College of Architecture and Urban Planning, Fujian University of Technology, Fuzhou 350000, China; fafulqy@outlook.com; 4 College of Forestry, Fujian Agriculture and Forestry University, Fuzhou 350000, China; yuyky@126.com

**Keywords:** urban green space, design intensity, restorativeness, preference, eye-tracking

## Abstract

Recent research has demonstrated that landscape design intensity impacts individuals’ landscape preferences, which may influence their eye movement. Due to the close relationship between restorativeness and landscape preference, we further explore the relationships between design intensity, preference, restorativeness and eye movements. Specifically, using manipulated images as stimuli for 200 students as participants, the effect of urban green space (UGS) design intensity on landscapes’ preference, restorativeness, and eye movement was examined. The results demonstrate that landscape design intensity could contribute to preference and restorativeness and that there is a significant positive relationship between design intensity and eye-tracking metrics, including dwell time percent, fixation percent, fixation count, and visited ranking. Additionally, preference was positively related to restorativeness, dwell time percent, fixation percent, and fixation count, and there is a significant positive relationship between restorativeness and fixation percent. We obtained the most feasible regression equations between design intensity and preference, restorativeness, and eye movement. These results provide a set of guidelines for improving UGS design to achieve its greatest restorative potential and shed new light on the use of eye-tracking technology in landscape perception studies.

## 1. Introduction

Currently, 55% of the world’s population lives in cities, and with the progressive urbanization this number is supposed to increase to 70% by 2050 [1]. As more and more people come to live in urban areas, the quality and sustainability of urban living environments become more and more important, and the topic has caught much attention. This is especially true in China, which is a rapidly developing country in which hundreds of thousands of people move into urban areas every year. Urban areas are not only centers of economic and social development but also primary causes of major environmental issues [2,3]. For instance, urban expansion resulted in biodiversity reduction, greenhouse effect caused by urban CO_2_ emission, urban sewage discharge led to water pollution, etc.

It is widely known that urban green space (UGS) plays a vital role in strengthening urban biodiversity and sustainability [4,5,6]. UGS can not only make urban ecosystems more sustainable (by providing oxygen, maintaining water and soil, reducing heat island effects, etc.) but also contribute directly to human health [7]. Some studies have shown that people can effectively recover from the negative health effects of stressful urban life by visiting green spaces [8,9]. In other words, UGS is perceived as a place with the potential to mitigate the negative psychophysiological impacts of densely built urban environments [5,10]. In addition, studies also have shown that individuals’ favorite place can provide psychological restorativeness [11,12,13] and that people were better able to recover from psychological fatigue and various negative emotions associated with stress in their preferred environment [11]. Several recent studies have replicated the finding that people’s preference for an environment can effectively improve the health benefits of that place [14,15,16]. However, most of the studies on green space health benefits have been conducted in Western countries, while only a few studies have been conducted in Chinese settings and against the Chinese cultural background [17]. For example, an extensive systematic review [18] included mainly European, Scandinavian, and US-based studies, with no studies in Chinese settings. Considering that preferences are closely related to restorativeness and that China’s urbanization has impacted human health and well-being by isolating human beings from the natural environment and through the pressure and anxiety brought by urban life [19], it is becoming increasingly important to know how to design UGS in Chinese circumstances to improve public preference for it, thereby encouraging more visits to UGS and improving restorative perception simultaneously.

Additionally, previous studies have mostly focused on landscape types, such as urban and natural, or visual aspects such as the presence or quantity of certain natural elements and have not explored the effects of different landscape design interventions to foster restorative perceptions in a space. This means current research makes limited contributions to direct design guidelines for UGS to reduce individuals’ stress. Moreover, eye movements are a normal aspect of daily visual perception, which is fundamental to landscape perception. Previous studies have suggested that analysis of eye movements can provide new insights into the research of restorative environments [20], and the eye metrics have been verified as valid in predicting restorative effects [21]. In this context, the present research expanded on prior studies by introducing the concept of landscape design intensity, describing the extent of design interventions, and evaluating the effects of landscape design intensity on landscape preference, restorativeness and eye movements, ultimately leading to a design guideline for restorative urban green environments and shedding new light on eye movement and restorative effects.

### 1.1. Landscape Design Intensity

The extent of design intervention can be captured as landscape design intensity (LDI), which was first proposed by Xu et al. [22], defined as “the amount of the original landscape changed and the degree of artificiality of added elements to the landscape by design”. This definition focused more on the change in landscapes from original state to final state over time. However, in UGS areas, people usually know little about the original state of a landscape. Therefore, in this study we modified this definition or LDI to cover number of different landscape elements used, the complexity of different landscape elements in a configuration, and the extent of landscape maintenance requirements in the present UGS landscape. Landscape design intensity was regarded as relative to preference because it has been found to be significantly related to visual aesthetic quality [22]. Landscapes with proper design intensity may improve visual aesthetic quality, thereby promoting recreational activities, attracting tourists [23] and contributing to human mental health [24]. Xu et al. demonstrated that people prefer natural or restored landscapes with moderate levels of design intensity [22]. However, their study did not focus on UGS landscape. An accruing body of research suggests that natural environments have better restorative outcomes than human-made urban environments [25,26,27,28]. As an important part of urban environments, UGS still has some characteristics of human-made urban environments (e.g., some structures, large hard surface paving area, some pruned plants etc.). Therefore, there is still a big difference between natural landscapes and UGS landscapes, which may result in a different landscape preference and restorativeness result. Another similar study that did focus on UGS shows that the density of green infrastructure is positively correlated with preference, but across moderate or high vegetation density environments, increases in density yielded only slight increases in landscape preference [29]. However, this study only focused on effects of different levels of vegetation density on preference. It is well known that the design of UGS should not be limited to plant elements, but should also consider the design of the overall environment. In this light, there is a need for further exploration of the relationship between design intensity and preference for urban green space, as well as its relation with restorativeness and its benefits for the design process.

### 1.2. Landscape Preference in Relation to Restorativeness

People’s preference for environment is a kind of subjective psychological evaluation. It is a judgment formed by a series of psychological reactions such as cognition, emotion and meaning after an individual experiences the environment through visual, auditory and other senses. The restorativeness of UGS mainly includes its effects of reducing stress and anxiety [30], preventing mental fatigue and depression [31,32], and improving attention and mood [33]. Two broadly accepted theoretical perspectives, Attention Restoration Theory (ART) [31] and Stress Reduction Theory (SRT) [34], are relevant to the restorative effects of UGS. ART maintains that prolonged use of direct attention leads to mental fatigue, but that environments with all four characteristics, namely “being away, fascination, extent, and compatibility” are more likely to simulate individuals’ indirect attention, thereby potentially restoring their attention capabilities [31]. Based on ART, Hartig and his colleagues [35,36] developed the Perceived Restorativeness Scale (PRS) to measure restorative effects of natural environments, and a lot of research with regards to this topic has been conducted with the PRS. From a different perspective, the SRT holds that when people are exposed to UGS, there will be positive shifts in emotional state, such as blocking out pessimistic thoughts or turning a bad mood into a good one and reducing stress [34]. As the PRS has been used broadly, the present study also measured the restorative effects of UGS compared to the PRS.

Previous studies have indicated that people prefer natural landscapes to urban ones, especially parks or large grasslands [31,37], and that they prefer brighter, more visually diverse, natural landscapes that contrast artificial environments [38]. In addition, numerous studies have revealed a significant positive relationship between landscape preference and perceived restorativeness, and natural environments are perceived as more restorative than urban environments [13,14,38,39]. This also means that with increasing preference for UGS, individuals’ perceived restorativeness will also increase. Other studies on restorative environments have mostly focused on certain elements in landscape features such as water or plants [37,40] or landscape characteristics such as naturalness [41], biodiversity [41,42], familiarity, social context, and perceived security [43] in relation to restorative potential. For example, Hoyle et al. [42] surveyed 1141 respondents who walked through plantings and found that people perceived higher restorativeness with moderately natural or the most natural planting structure than with the least natural structure. Kang and Kim [44], using two variables—natural/built scene and close/distant view—found that regardless of difference in distance, natural landscapes were perceived as more restorative than urban environments and visual aesthetics and complexity contributed to the restorative effect. Although these existing studies have dealt with various predictors for restorative effects and have broadly established the significant relationship between landscape preference and restorativeness, their results provide limited guidance for landscape architects when deciding between landscapes using different design interventions. It is necessary to consider the role of design intensity in landscape preference and restorativeness because we assume that they can provide such guidance, for example pointing us toward using a design intervention that leads to a more restorative landscape. The possibility of linking landscape design intensity with preference and restorativeness will allow landscape architects and UGS decision makers and managers to measure the outcomes of different design interventions and make better decisions.

### 1.3. Eye-Tracking Analysis in Landscape Perception Research

To date, landscape perception studies have mainly relied on subjective approaches, such as questionnaires and in-depth interviews. Nowadays, however, eye-tracking technology can provide a more accurate method to study this topic in greater detail. Eye trackers can record people’s eye movement trajectory quickly and accurately as the people observe objects, thereby providing an opportunity for researchers to explore the human perception process or mental activity [45]. Eye movements can also allow us to explore the underlying processes of landscape appreciation and attention recovery. Several studies have shown the possibility of using eye-tracking technology in landscape preference and restorative environment study [44,45,46,47]. For instance, Noland and his colleagues [45] and Dupont and her colleagues [46] have found a significant correlation between individuals’ preference and eye movements (include eye fixations and their duration). The other similar research results also demonstrated that higher ornamental landscape attracted more attention and interest from observers than the lower ornamental landscape. Additionally, Kang and Kim [44] have indicated that landscape types with different restorative effects differ in scan path length. Among the several types of eye movements observed in humans, the metrics of fixations and saccades (moments in which the eye moves jerkily, rather than smoothly, as when following an object in motion) have been widely considered [48,49]. It means these eye-tracking metrics (eye fixations, fixation duration and saccades) are likely to be the predictors of landscape preferences. Furthermore, Valtchanov and Ellard compared people’s eye movements when they watched pictures of the city and of the natural landscape, and they found that the natural landscape pictures yielded more fixation time [50]. In contrast, Berto found that people watched the urban landscape with larger saccade distance and longer fixation time than natural landscape photos [21]. The contradiction here may make people doubtful. Further research is needed to explain these results. However, these studies can provide a good research paradigm for using eye-tracking technology in landscape perception research. Although the literature has provided some understanding of the relationship among landscape preference, restorativeness, and eye movements, it is still relatively uncommon to apply eye-tracking technology in studies focusing on landscape preference and restorativeness [51], especially in Chinese settings. Therefore, this study adopted an eye-tracking approach to explore the relationship between landscape design intensity, landscape preference, and restorativeness with Chinese UGS scenes in order to evaluate landscape design interventions more accurately and provide robust, data-driven support for the development of landscape preference and restorative environment theory. It can also help designers understand the effect of design intensity on landscape preference and restorativeness, and enrich their knowledge of design intensity so that they can choose the appropriate design intensity during the design process. In addition, using eye-tracking technology will develop new knowledge regarding landscape preference and restorative environments from the perspective of human physiological response (eye movement behavior) so that we can understand the interaction mechanism between landscape and human eye movement behavior.

### 1.4. Research Questions

This study used design intensity as a metric to describe the extent of design interventions in UGS and explored the relationship between landscape design intensity, landscape preference, and restorativeness in order to evaluate the outcome of different design interventions. Landscape images of UGS with different levels of design intensities (lowest, low, moderate, and high) were used as stimuli. In addition, an eye-tracking methodology was applied to generate new knowledge on restorative environments and to show the value of eye-tracking technology in landscape perception studies. We measured eye movements across landscapes with four levels of design intensity. The following three research questions were explored:

1. How does design intensity affect people’s eye movements, landscape preference and restorativeness?
2. What is the overall relationship among UGS design intensity preference, restorativeness, and eye movement?
3. Are there linear or curvilinear regressions relationship between design intensity and preference, restorativeness, and eye movement? Which equation works better?

## 2. Materials and Methods

### 2.1. Study Design

A photo-based approach to evaluation was used in this study, this method has been widely used by previous researchers [52], and its reliability has been generally accepted [53]. In addition, we adopted the method of editing photos to control the research variables, which provides better control over nonrelevant random variables (e.g., weather conditions and background features) [54] than do assessments of current UGS scenes. The study design included three parts. First, the participants were divided into five groups of UGS landscape randomly, and equally. Each of them could only observe landscape pictures in their own group. Every group of four pictures, representing four design intensities, was present on the monitor at the same time to create valid comparisons. Participants were asked to observe the monitor freely while being measured by an eye tracker. The four pictures were very similar with the exception of one or more relevant varying features to create different design intensities, which is helpful to control confounding variables and assure the comparability of the four pictures. In addition, the four pictures of the same size were randomly arranged in a 2 × 2 format in order to eliminate the influence of pictures’ position and size. Second, to obtain individuals’ preference from among the four pictures in each group, the Law of Comparative Judgment (LCJ) method was utilized, and respondents were asked to compare the four pictures and then give their preference ratings. Finally, respondents were asked to give PRS ratings for every picture randomly shown on the laptop. This study was approved by the college.

### 2.2. Study Stimulus

**Figure 1:**
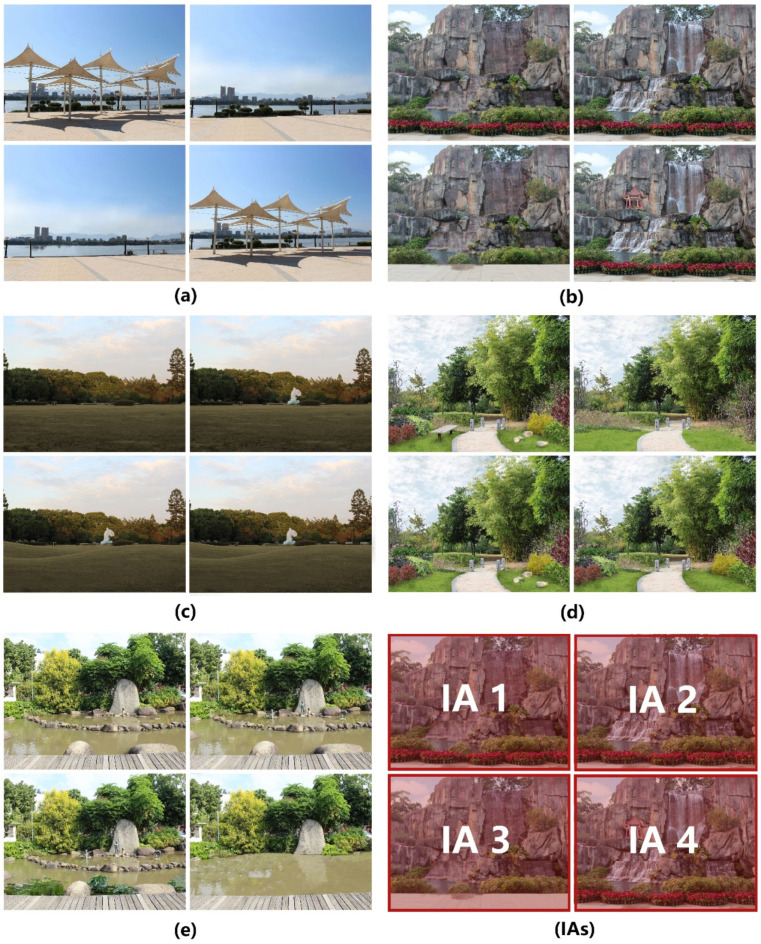
Stimulus Photographs and IAs’ example. (**a**–**e**): the five sets of experimental photos. (**IAs**): an example of four IAs.

A total of five urban green space images was selected for the research from a photo bank, all taken in summer. These five pictures were selected based on (1) how typically they represent the most common Chinese UGS scenes, (2) how free they were from distracting elements such as intense color scheme or pattern, and (3) how feasibly other landscape elements could be incorporated or current landscape elements removed in the picture. Based on the five photos, three additional levels of design intensity for each image were created using Photoshop CS6 to remove or synthesize landscape elements, such as stones, pavilions, and sculptures. The inserted or removed landscape elements were geared to the conditions in the original pictures. This procedure resulted in a total of 20 simulations. These photos were then reviewed by two senior researchers with much experience in visual assessment studies in order to ensure their rationality and realism. In addition, 10 experts were invited to classify these photos into four design intensity categories, from lowest to high. Finally, the pictures in each set were randomly arranged with two pictures vertically and two pictures horizontally on a 2126 px width and 1594 px height screen. Each picture represents one interest area (IA), and there are four interest areas on the screen at a given time. The upper left photo was marked IA 1, the upper right photo IA 2, the bottom left photo IA 3, and the bottom right photo IA 4. Figure 1 shows the five experimental screens and an example of four IAs.

### 2.3. Eye-Tracking Apparatus and Metrics

A desktop eye tracker Eye-link 1000 plus (SR Research, Kanata, ON, Canada) [48] was used to record participants’ eye movements. The sampling rate of the apparatus was adjusted to 1000 Hz in the monocular (right eye) Pupil–CR recording mode. Images were displayed in the center of a 19-inch screen. A chin rest was used to fix participants’ heads in place. The distance between participants and the monitor was set at 55 cm [55].

For each IA on the screen, the eye movements metrics of dwell time percent, fixation percent, fixation count and first fixation visited interest area count were used. Dwell time reports the percentage of time spent dwelling on the current IA compared to the total time spent looking at a set of photographs. Fixation percent describes the percentage of all fixations in a set of photographs that fall in the current IA. Fixation count is the number of fixations in the current IA. First fixation visited interest area count reports the number of IAs visited before the first fixation on the current IA. This metric indicates the extent of the current IA attracting participants, which means the earlier participants focused on the current IA, the more attracting the current IA is. In order to more intuitively understand the order in which interest areas attracted peoples’ visual attention, we use the concept of visited ranking to substitute the first fixation visited interest area count; the first visited IA was scored 4, the second 3, and so on.

### 2.4. Participants

The participants were 92 males and 108 females affiliated with a university, who were recruited online. Most of them were between 20 and 23 years old. These students were enrolled in plant science, landscape architecture, economics, and social sciences, and over half of them were undergraduates. The 200 participants were assigned equally to five groups, with 40 participants per group, for the eye movement experiments, and were required to wear contact lenses instead of glasses if they needed corrective lenses [56]. All participants had normal or corrected-to-normal vision [57]. None of the participants suffered from vision-related illnesses or colorblindness. Because one male participant did not finish the eye movement experiment, his eye movement data were removed.

### 2.5. Procedure

**Figure 2:**
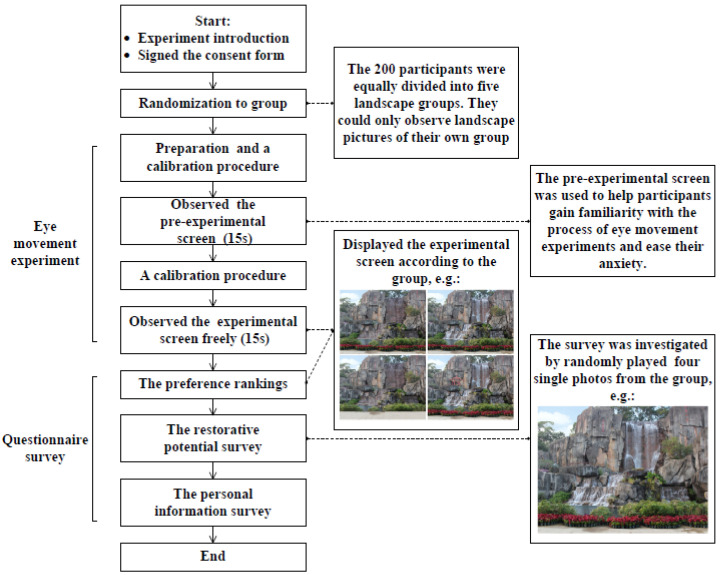
A flowchart of the experimental process.

The experimental process flowchart is illustrated in Figure 2. All participants were tested individually. Upon their arrival, participants were informed of the study’s purpose and procedures in detail and were given a consent form to read and sign. We would divide the participants into groups according to the requirements in the 2.1. Study design section above. On agreeing to participate in the experiment, the respondents were positioned in front of a monitor fitted with the eye tracker. During the experiment, the participants placed their heads on a chin rest to minimize their head movements and viewed a monitor placed 55 cm from the surface of their cornea. Each participant was then asked to follow a black circle on the monitor with their eyes to calibrate the eye tracker. Calibration was completed using a nine-dot calibration procedure that allowed the eye-tracking system to match the pupil-center/corneal reflections related to the specific x-y coordinates of the dot [55]. After the calibrating procedure was completed, the participants were presented with a pre-experiment image on the monitor, for 15 s. The pre-experiment image was used to help participants gain familiarity with the process of eye movement experiments and ease their anxiety to reduce potential errors in the eye movement data.

Following the pre-experiment, the participants performed another one-point calibration, and the experiment began with a screen (displayed four landscape photos at the same time) shown for 15 s, followed by a rating task that asked respondents to provide their preference rankings among the four landscapes shown on the laptop screen (1 = lowest ranking; 4 = highest ranking). After that, each of these four photos was presented on the laptop screen randomly and after viewing every picture, the respondents were asked to imagine themselves in the scene and respond to the restorative potential survey. The restorative potential was assessed using a four-item scale, which has been used in former studies with Chinese populations and identified to have good interclass reliability (range from 0.86 to 0.90) and validity [10]. The following four statements were presented: “Spending time in the scene gives me a good break from my day-to-day routine (Being away),” “The scene has sufficient content and structure that it can occupy my mind for a long period (Extent),” “My attention is attracted by the scene (Fascination),” and “I would like to stay here longer, as I can enjoy myself in this scene (Compatibility).” The respondents were asked to rate their agreement level for each statement on a Likert scale, from “fully disagree (1)” to “fully agree (7).” Additionally, the participants were asked to provide their personal information, including their age, gender, education level (undergraduate or postgraduate), and major.

### 2.6. Data Analysis

Data from the eye movement experiment were exported via data viewer software. Statistical analysis was carried out with SPSS 23.0 software(IBM Corp., Armonk, NY, USA).

According to the results of histogram, preference, restorative rating, and visited ranking met the requirement of approximately normal distributions, but dwell time percent, fixation percent, and fixation count did not. The data regarding preference, restorative rating, and the four eye-tracking metrics were analyzed using descriptive statistics. One-way ANOVA was conducted to compare respondents’ dwell time percent, fixation percent, and fixation count for various design intensities. Kruskal–Wallis one-way ANOVA (K sample) was used to test the differences from preference, restorative rating, and visited ranking between various design intensity. Then, the overall relationship between landscape design intensity, preference, restorative potential, and eye movement was computed using Spearman’s correlation analysis. Finally, we ran linear and curvilinear regressions to identify the most feasible model to describe the relationship among design intensity, preference, and restorative rating.

## 3. Results

### 3.1. Comparison of Eye-Tracking Metrics, Preference and Restorative Rating among Design Intensities

Table 1 presents the participants’ preference and restorative ratings regarding each IA. The images with high design intensities obtained the highest dwell time percent (30.63 ± 14.11), fixation percent (30.01 ± 12.25), fixation count (14.45 ± 5.99), and restorative rating (5.50[(1.25)). Similarly, the photos depicting UGS with lowest design intensity received the lowest dwell time percent (18.99 ± 8.94), fixation percent (19.25 ± 8.74), fixation count (9.40 ± 4.63), and restorative rating (4.75(2.25)). Additionally, the high and moderate design intensity landscapes received the same highest preference score (3.00(2.00)). The images with high and low design intensities obtained the same highest visited ranking (3.00(2.00)). Combined analyses of data from, preference, eye-tracking metrics (except visited ranking), and restorative ratings had an overall upward trend with the improvement of design intensity.

The results of the Kruskal–Wallis test and one-way ANOVA revealed that eye-tracking metrics, preference, and restorative ratings significantly differed across design intensity.

### 3.2. Correlation among Design Intensity, Preference, Restorative Rating, and Eye Movement Metrics

The correlation analysis results between landscape design intensity, preference, restorative ratings, and eye-tracking metrics are illustrated in Table 2. There were significant positive correlations between landscape design intensity and preference (r = 0.475, *p* ≤ 0.001), design intensity and restorative ratings (r = 0.193, *p* ≤ 0.001), and design intensity and the four eye-tracking metrics. In terms of preference, all relationships were significant except for between preference and visited ranking. Furthermore, restorative ratings were found to significantly correlate with fixation percent (r = 0.075, *p* ≤ 0.05). There were also significant positive correlations between dwell time percent and fixation percent (r = 0.942, *p* ≤ 0.001), dwell time percent and fixation count (r = 0.885, *p* ≤ 0.001), dwell time percent and visited ranking (r = 0.255, *p*≤ 0.001), fixation percent and fixation count (r = 0.936, *p* ≤ 0.001), between fixation percent and visited ranking (r = 0.275, *p* ≤ 0.001), and between fixation count and visited ranking (r = 0.248, *p*≤ 0.001).

These results would seem to suggest that higher landscape design intensifies higher preferences, restorative ratings, dwell time percent, fixation percent, fixation count, and visited ranking. The higher preference may cause the higher restorative rating, dwell time percent, fixation percent, and fixation count. There were positive correlations between the four eye-tracking metrics.

### 3.3. The Relationship between Design Intensity, Eye-Tracking Metrics, Preference, and Restorative Rating

According to the 3.2, the relationships between design intensity, preference, and restorativeness were significantly positively correlated. It is also possible that curvilinear models might explain these relationships. To investigate these possibilities, we ran linear—and then curvilinear—regressions to identify the most feasible model to describe the relationship. Table 3 displayed the values of the Adjusted R-Squared (Adj-R^2^) and *p* of different statistical models. For the relationship between design intensity and preference, the quadratic curve model fit better with the data with the higher Adj-R^2^ (0.236 vs. 0.225). Therefore, there is the regression equation of preference = 0.698 + 1.091 × design intensity − 0.123 × design intensity^2^. For the relationship between design intensity and restorative rating, the Adj-R^2^ value of the linear model was better than the quadratic curve model (0.044 vs. 0.043). The regression equation of restorative rating = 4.357 + 0.240 × design intensity. Similarly, the linear model between preference and restorative rating also had a better Adj-R^2^ value (0.015 vs. 0.014). The linear regression equation of restorative rating = 4.603 + 0.142 × preference.

Additionally, the most feasible regression equation between design intensity and four eye-tracking metrics were dwell time percent = 15.002 + 3.754 × design intensity (Adj-R^2^ = 0.129); fixation percent = 15.726 + 3.435 × design intensity (Adj-R^2^ = 0.129); fixation count = 7.749 + 1.618 × design intensity (Adj-R^2^ = 0.110); and visited ranking = 1.545 + 0.744 × design intensity − 0.121 design intensity^2^(Adj-R^2^ = 0.029).

The data reported here appear to support that, except for the most feasible regression equations of design intensity and preference, and design intensity and visited ranking were quadratic regression equations, while the others were linear equations.

## 4. Discussion

### 4.1. Design Intensities in Relation to Preference and Restorativeness

The present study introduced the concept of landscape design intensity and revealed that it positively affects preference and restorativeness, meaning that with increased landscape design intensity, the participants’ preferences and perceived restorativeness for UGS scenes will also increase. The regression equations are preference = 0.698 + 1.091 × design intensity − 0.123 × design intensity^2^ and restorative rating = 4.603 + 0.142 × preference. Based on our definition of landscape design intensity, it is clear that UGS scenes with high design intensity may have a greater number and variety of landscape elements, a more complicated configuration, or certain modeling shrubs that need higher maintenance. With such increases in design intensity, UGS scenes may become more attractive and interesting. Therefore, we can postulate that a landscape with high design intensity provides subjects with more environmental information to explore, thereby increasing individuals’ preference for the scenes. Previous studies also support our inference [10,29,58]. Wang et al. used manipulated images to explore the effects of UGS characteristics on aesthetic preference and concluded that aesthetic preference was enhanced by increasing trees or flowers [10]. In Kaplan’s research concerning workplace scenes, they similarly found that a landscape with vegetation can enhance employees’ preference and satisfaction [58]. Additionally, we found that the optimal relationship between design intensity and preference is a quadratic function model. This result is consistent with previous studies that found that the relationship between complexity and preference is an inverted U-shaped curve. It may reflect that the excessive design intensity lead to the decline of landscape preference. The excessive design intensity likely provides a large amount of environmental information, resulting in people’s burden on information processing, thus weakening the preference. However, we did not adopt excessive design intensity, which is not consistent with the actual landscape design (designers would not use excessive design intensity due to their professional quality). Therefore, more research is required to explore why excessive design intensity has a negative effect on preference.

Additionally, the positive effect of landscape design intensity on restorativeness was similar to previous studies, suggesting that landscape with an increasing number of trees contributed to landscape complexity [32] and thereby restorativeness [59]. This is in line with Kang and Kim [44], who demonstrated that landscape complexity had a statistically significant restorative effect relationship. This result may be explained with reference to Attention Restoration Theory; UGS scenes with high design intensity usually include beautiful landscape content and stimulate participants’ involuntary attention (fascination). With the increased design intensity, more landscape elements (such as trees, flowers and sculptures) appear, and individuals feel like they are entering a new world to enjoy nature or human art as a break from the daily routine (being away). In these circumstances, people may immerse themselves, as the scenes may meet their need for rest from activity (compatibility). Additionally, landscapes with high design intensity provide information, which may inspire participants’ imagination and their will to explore (extent).

The significant positive relationship between preference and restorativeness in the present study is not surprising because many studies have confirmed this positive correlation [10,11,14,16]. The most feasible regression equation is the restorative rating = 4.603 + 0.142 × preference. Therefore, landscape preference was identified as a positive predictor of restorativeness. As design intensity has a significant positive correlation with preference and restorativeness, preference may also have an indirect role between landscape design intensity and restorativeness. UGS with high design intensity makes scenes complex and rich, stimulating individuals’ preference and enhancing respondents’ perceived restorativeness. Thus, approaches used to increase landscape design intensity may improve participants’ preference and enhance direct and indirect restorative effects.

### 4.2. Eye-Tracking Data and Their Relation with Design Intensity, Preference, and Restorativeness

By analyzing participants’ eye-tracking data across landscapes with four different design intensities, we found significant differences. Additionally, dwell time percent, fixation percent, fixation count, and visited ranking were positively correlated with design intensity, meaning that with increased design intensity, participants spend higher dwell time percent and fixation percent and exhibit more fixations and focus earlier on the scenes. The relevant regression equations are dwell time percent = 15.002 + 3.754 × design intensity; fixation percent = 15.726 + 3.435 × design intensity; fixation count = 7.749 + 1.618 × design intensity; and visited ranking = 1.545 + 0.744 × design intensity − 0.121 design intensity^2^. Landscapes with high design intensity result in high complexity and richness, and a complex landscape provides a large amount of information to be processed [60] and a larger interest value of the stimulus [61]. Previous study on the complexity of landscapes has demonstrated that the more complex and informative an urban landscape is, the more extensive and dispersed exploration will be [62]. Therefore, participants will spend more time focusing more on high-design-intensity scenes, resulting in higher eye-track metrics values.

The results also revealed that preference has a significant positive relationship with dwell time percent, fixation percent, and fixation count. This is also consistent with previous findings [47,63,64]. Some existing research also demonstrated that green landscapes with higher ornamental value attracted more attention and interest from observers than did landscapes with lower ornamental value [65]; therefore, we may infer that landscapes with higher design intensity will result in a higher preference and elicit more visual exploration.

Additionally, restorativeness was found to have a significant positive relationship with fixation percent but a nonsignificant relationships with dwell time percent, visited ranking, and fixation count. Nordh et al. also found no correlation between the number of fixations and restorativeness likelihood ratings, and they concluded that spending time at landscape components can be both negatively and positively associated with restorativeness likelihood [66]. Another study found that high dwell time is related to the complexity of content, as complex content may need more cognitive effort to perceive and interpret [67], which may consume more attention and result in attention fatigue. Thus, we speculate that dwell time percent, fixation count and fixation percent are good predictors for restorativeness. However, they could function negatively, positively, or both negatively and positively. More studies are necessary to provide more insight into this.

### 4.3. Implications for UGS Design

The results of this study demonstrated that landscape design intensity has a positive impact on landscape preference, restorativeness, and eye movements. Higher design intensity will promote preference and facilitate restorativeness in both direct and indirect ways. These findings are useful for landscape architects and practitioners interested in improving UGS design and helping it reach its greatest restorative potential. To improve the restorative outcomes of UGS scenes, based on the present study, increasing the design intensity of UGS is a good choice. There are several ways to improve design intensity. Designers may choose to use several landscape elements to make scenes look attractive or choose to arrange landscape content in a complex structure, or even alter the topography of the landscape scenes. Additionally, although many designers do not choose excessive design intensity as they have good professional qualities (e.g., visual aesthetics and spatial organization ability), we should be aware that excessive design intensity may have a negative impact on preference, thus restorativeness. Simultaneously, when designing UGS scenes, other aspects, such as ecological factors, biodiversity factors, and budgets, should also be considered.

Additionally, the eye-tracking results demonstrated the great potential of this technology in comparing respondents’ reactions to landscape scenes with different design interventions. Eye-tracking technology could also be used to compare different participants’ reactions to a particular landscape scene. Therefore, eye-tracking technology may help landscape architects examine the design outcomes of UGS and gather a range of perceptions of landscape quality from different user groups, thereby promoting a better design process.

### 4.4. Limitations and Further Study

Several limitations of this study should be addressed. First, landscape preference research suggests that demographic differences among the respondents (e.g., gender, age, education, occupation, living environment) have a considerable impact on individual landscape preferences [40,68,69]. In this study, only students and teachers were recruited as participants; therefore, it is necessary to involve a wider range of respondent demographics and explore the demographic variables related to the relationship between design intensity and landscape preference in future studies. Second, the stimulus was displayed on the screen for 15 s. Some participants may have felt that this was too much time, and thus a 15-s display could cause irrelevant eye movements and affect the results of this research. Therefore, further studies should allow participants to control how long they view images to improve their result’s accuracy. Another limitation in the present study is that we did not track detailed restorative components in the stimulus, resulting in limited guidance for restorative environments. Further research using eye-tracking technology to track the restorative elements and restorative landscape composition would help overcome this limitation.

## 5. Conclusions

This study has introduced the concept of design intensity and examined the association between design intensity, landscape preference, and perceived landscape restorativeness, and assessing eye-tracking metrics based on manipulating images. This approach enriched current research on landscape preference and eye tracking and added new insights to the existing research. The results demonstrated that landscape design intensity could positively contribute to preference and restorativeness, as well as eye-tracking metrics, including dwell time percent, fixation percent, fixation count, and visited ranking; this generally supports previous arguments that participants’ preference generally increases with higher landscape complexity (design intensity covers the complexity). According to the regression equation (preference = 0.698 + 1.091 ×design intensity − 0.123 × design intensity^2^), it indicated that appropriately increased LDI can improve preference and excessive LDI may have a negative impact. However, we did not adopt excessive LDI in this study, which is not consistent with the actual landscape design (designers would not use excessive design intensity due to their professional quality). It is implicated that more research is required to explore why excessive LDI has a negative effect on preference, and the judgment of the highest threshold and the input–output ratio. Consistent with previous studies, we found that preference was positively related to restorativeness and some eye-tracking metrics (include dwell time percent, fixation percent, and fixation count). Additionally, the results indicated that there are significant relationships, negative or positive, between restorativeness and dwell time percent and fixation percent. Although a previous study did not find a significant correlation between restorative and eye movement, some other studies have found that eye movement has two different effects on restorativeness, positive or negative. The results may be related to the attention consumed during eye movement. We need further investigation to explain this phenomenon. Despite some limitations in this study, these results provide valuable cues for UGS design to improve the restorative potential and generate new knowledge of the association between preference, restorativeness and eye movements. It can help researchers further investigate the associations between landscape design intensity, eye movements, and other indicators (e.g., landscape complexity and coherence). For practitioners, incorporating the findings of the effects of landscape elements on complexity and preference into the design process could lead to improved designs for future urban parks in China and other countries. Moreover, the study also demonstrated the potential of using eye-tracking technology in landscape preference, and restorative environments research.

## Figures and Tables

**Table 1:** Eye-tracking metrics, preference and restorative ratings of the changes resulting from the different design intensity.

		Design Intensity				F/H	*p*
		Lowest(N = 39)	Low(N = 39)	Moderate(N = 39)	High(N = 39)		
Eye-tracking metrics	Dwell time percent	18.99 ± 8.94	22.65 ± 8.50	25.27 ± 10.91	30.63 ± 14.11	40.554 ^‡^	0.000
	Fixation percent	19.25 ± 8.74	22.97 ± 8.50	25.04 ± 9.85	30.01 ± 12.25	40.504 ^‡^	0.000
	Fixation count	9.40 ± 4.63	11.16 ± 4.53	12.17 ± 5.23	14.45 ± 5.99	33.711 ^‡^	0.000
	Visited ranking *	2.00(2.00)	3.00(2.00)	2.00(2.00)	3.00(2.00)	58.736 ^†^	0.000
Preference *		1.00(1.00)	2.00(1.00)	3.00(2.00)	3.00(2.00)	192.123 ^†^	0.000
Restorative ratings *		4.75(2.25)	5.00(2.00)	5.00(1.75)	5.50(1.25)	30.464 ^†^	0.000

Note: Values: means ± standard deviations. *: median (interquartile range); ^†^: Kruskal–Wallis test; ^‡^: One-way ANOVA.

**Table 2:** Correlations between design intensity, preference, eye-tracking metrics, and restorative ratings (N = 796).

Variable		2	3	4	5	6	7
1	Design intensity	0.475 ***	0.193 ***	0.340 ***	0.341 ***	0.321 ***	0.141 ***
2	Preference	-	0.116 ***	0.147 ***	0.148 ***	0.142 ***	0.002
3	Restorative rating	0.116 ***	-	0.063	0.075 *	0.045	0.026
4	Dwell time percent	0.475 ***	0.063	-	0.942 ***	0.885 ***	0.255 ***
5	Fixation percent	0.148 ***	0.075 *	0.942 ***	-	0.936 ***	0.275 ***
6	Fixation count	0.142 ***	0.045	0.885 ***	0.936 ***	-	0.248 ***
7	Visited ranking	0.002	0.026	0.255 ***	0.275 ***	0.248 ***	-

Note: *: *p* ≤ 0.05, ***: *p* ≤ 0.001.

**Table 3:** Regression model fitness for the relationship between design intensity, preference, and restorative rating.

Independent	Dependent	Model	Adj-R^2^	F	*p*	Constant	b1	b2
Design intensity	Preference	Linear	0.225	231.219	0.000	1.314 ***	0.475 ***	
		Quadratic	0.238	123.591	0.000	0.698 ***	1.091 ***	−0.123 ***
	Restorative rating	Linear	0.044	38.000	0.000	4.357 ***	0.240 ***	
		Quadratic	0.043	19.004	0.000	4.408 ***	0.190	0.010
	Dwell time percent	Linear	0.129	119.151	0.000	15.002 ***	3.754 ***	
		Quadratic	0.130	60.213	0.000	17.141 ***	1.615	0.428
	Fixation percent	Linear	0.129	118.508	0.000	15.726 ***	3.435 ***	
		Quadratic	0.129	56.629	0.000	17.287 ***	1.874	0.312
	Fixation count	Linear	0.110	99.044	0.000	7.749 ***	1.618 ***	
		Quadratic	0.109	49.756	0.000	8.408 ***	0.958	0.132
	Visited Ranking	Linear	0.019	16.037	0.000	2.148 ***	0.141 ***	
		Quadratic	0.029	12.868	0.000	1.545 ***	0.744 ***	−0.121 **
Preference	Restorative rating	Linear	0.015	12.843	0.000	4.603 ***	0.142 **	
		Quadratic	0.014	6.440	0.002	4.553 ***	0.192	−0.010

Note: **: *p* ≤ 0.01, ***: *p* ≤ 0.001.

## Data Availability

The data is available from the corresponding authors on reasonable request.
